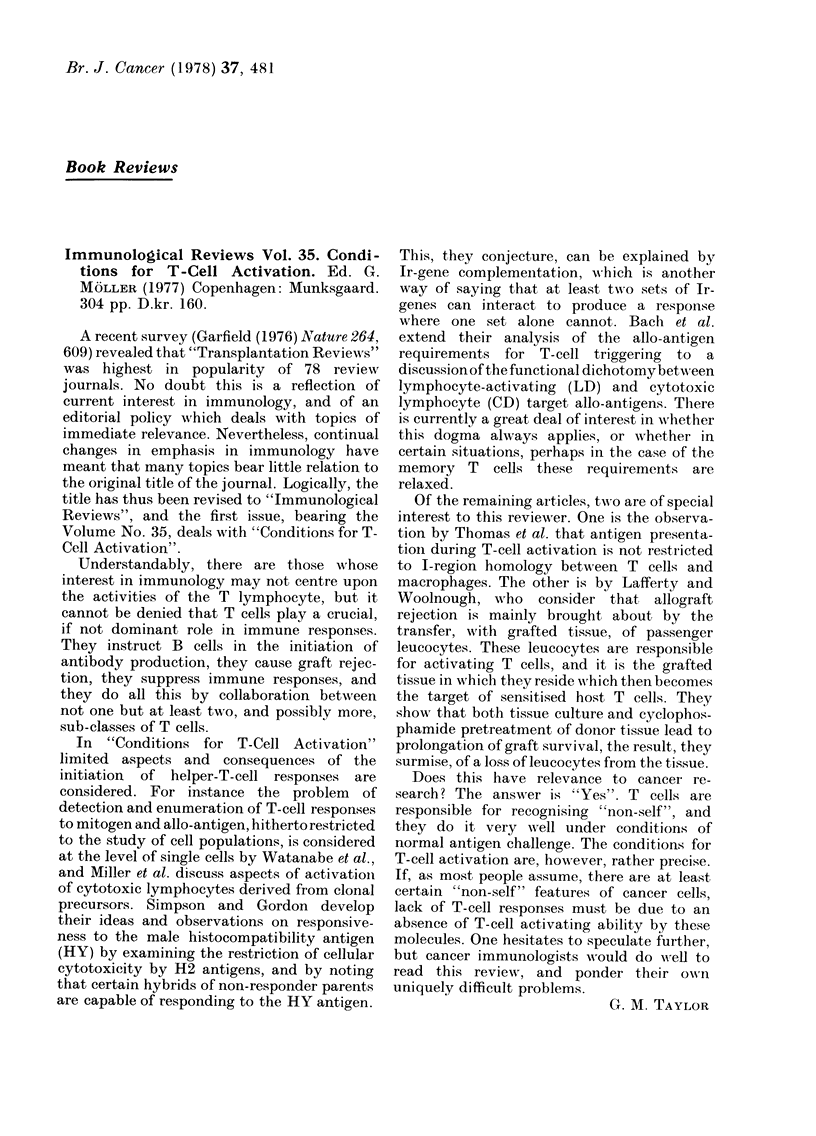# Immunological Reviews Vol. 35. Conditions for T-Cell Activation

**Published:** 1978-03

**Authors:** G. M. Taylor


*Br. J. Cancer* (1978) **37**, 481

### *Book Reviews*

**Immunological Reviews Vol. 35. Conditions for T-Cell Activation.** Ed. G. Möller (1977) Copenhagen: Munksgaard. 304 pp. D.kr. 160.

A recent survey (Garfield (1976) *Nature 264*, 609) revealed that "Transplantation Reviews" was highest in popularity of 78 review journals. No doubt this is a reflection of current interest in immunology, and of an editorial policy which deals with topics of immediate relevance. Nevertheless, continual changes in emphasis in immunology have meant that many topics bear little relation to the original title of the journal. Logically, the title has thus been revised to "Immunological Reviews", and the first issue, bearing the Volume No. 35, deals with "Conditions for T-Cell Activation".

Understandably, there are those whose interest in immunology may not centre upon the activities of the T lymphocyte, but it cannot be denied that T cells play a crucial, if not dominant role in immune responses. They instruct B cells in the initiation of antibody production, they cause graft rejection, they suppress immune responses, and they do all this by collaboration between not one but at least two, and possibly more, sub-classes of T cells.

In "Conditions for T-Cell Activation" limited aspects and consequences of the initiation of helper-T-cell responses are considered. For instance the problem of detection and enumeration of T-cell responses to mitogen and allo-antigen, hitherto restricted to the study of cell populations, is considered at the level of single cells by Watanabe *et al.*, and Miller *et al.* discuss aspects of activation of cytotoxic lymphocytes derived from clonal precursors. Simpson and Gordon develop their ideas and observations on responsiveness to the male histocompatibility antigen (HY) by examining the restriction of cellular cytotoxicity by H2 antigens, and by noting that certain hybrids of non-responder parents are capable of responding to the HY antigen. This, they conjecture, can be explained by Ir-gene complementation, which is another way of saying that at least two sets of Ir-genes can interact to produce a response where one set alone cannot. Bach *et al.* extend their analysis of the allo-antigen requirements for T-cell triggering to a discussion of the functional dichotomy between lymphocyte-activating (LD) and cytotoxic lymphocyte (CD) target allo-antigens. There is currently a great deal of interest in whether this dogma always applies, or whether in certain situations, perhaps in the case of the memory T cells these requirements are relaxed.

Of the remaining articles, two are of special interest to this reviewer. One is the observation by Thomas *et al.* that antigen presentation during T-cell activation is not restricted to I-region homology between T cells and macrophages. The other is by Lafferty and Woolnough, who consider that allograft rejection is mainly brought about by the transfer, with grafted tissue, of passenger leucocytes. These leucocytes are responsible for activating T cells, and it is the grafted tissue in which they reside which then becomes the target of sensitised host T cells. They show that both tissue culture and cyclophosphamide pretreatment of donor tissue lead to prolongation of graft survival, the result, they surmise, of a loss of leucocytes from the tissue.

Does this have relevance to cancer research? The answer is "Yes". T cells are responsible for recognising "non-self", and they do it very well under conditions of normal antigen challenge. The conditions for T-cell activation are, however, rather precise. If, as most people assume, there are at least certain "non-self" features of cancer cells, lack of T-cell responses must be due to an absence of T-cell activating ability by these molecules. One hesitates to speculate further, but cancer immunologists would do well to read this review, and ponder their own uniquely difficult problems.

G. M. Taylor